# Di-μ-hydroxido-bis­[hemiaqua­(*N*,*N*,*N*′,*N*′-tetra­methyl­ethane-1,2-diamine)­copper(II)] bis­(tetra­fluoridoborate)

**DOI:** 10.1107/S1600536812021836

**Published:** 2012-05-19

**Authors:** Jaroslava Haníková, Juraj Kuchár, Zdeněk Trávníček, Juraj Černák

**Affiliations:** a Department of Inorganic Chemistry, Institute of Chemistry, P. J. Šafárik University in Košice, Moyzesova 11, 041 54 Košice, Slovakia; b Department of Inorganic Chemistry, Faculty of Science, Palacký University, Tř. 17. listopadu 12, 771 46 Olomouc, Czech Republic

## Abstract

The title compound, [Cu_2_(OH)_2_(C_6_H_16_N_2_)_2_(H_2_O)](BF_4_)_2_, consists of dinuclear centrosymmetric [Cu_2_(OH)_2_(tmen)_2_(H_2_O)]^2+^ complex cations (tmen = *N*,*N*,*N*′,*N*′-tetra­methyl­ethane-1,2-diamine) and tetra­fluoridoborate anions. In the cation, the Cu^II^ atom shows a slightly distorted square-pyramidal coordination geometry provided by a pair of μ-OH^−^ anions and by the N atoms of a chelate tmen ligand in the basal plane. The apical position is statistically occupied by the O atom of a half-occupancy water mol­ecule. The F atoms of the anion are disordered over three sets of sites with occupancies of 0.598 (9):0.269 (6):0.134 (8). The crystal packing is governed by ionic forces as well as by O—H⋯F hydrogen bonds.

## Related literature

For the structures of related copper(II) complexes, see: Haníková *et al.* (2012 ▶); Handley *et al.* (2001 ▶); Černák *et al.* (2010 ▶). For additional structural analysis, see: Addison *et al.* (1984 ▶).
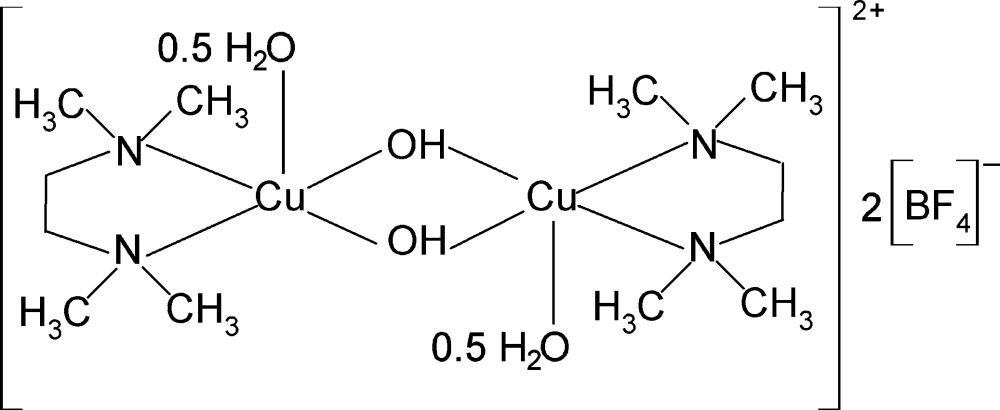


## Experimental

### 

#### Crystal data

[Cu_2_(OH)_2_(C_6_H_16_N_2_)_2_(H_2_O)](BF_4_)_2_
*M* *_r_* = 585.32
Monoclinic, 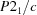

*a* = 7.5878 (3) Å
*b* = 14.7081 (3) Å
*c* = 11.4164 (3) Å
β = 109.174 (4)°
*V* = 1203.41 (6) Å^3^
*Z* = 2
Mo *K*α radiation
μ = 1.85 mm^−1^
*T* = 103 K
0.45 × 0.34 × 0.25 mm

#### Data collection

Oxford Diffraction Xcalibur Sapphire2 diffractometer
Absorption correction: numerical [Clark & Reid (1995 ▶) in *CrysAlis PRO* (Oxford Diffraction, 2009 ▶)] *T*_min_ = 0.475, *T*_max_ = 0.630
9405 measured reflections
2118 independent reflections
1916 reflections with *I* > 2σ(*I*)
*R*_int_ = 0.017

#### Refinement

*R*[*F* ^2^ > 2σ(*F* ^2^)] = 0.046
*wR*(*F* ^2^) = 0.143
*S* = 1.12
2118 reflections
168 parameters
1 restraint
H-atom parameters constrained
Δρ_max_ = 1.01 e Å^−3^
Δρ_min_ = −0.61 e Å^−3^

### 

Data collection: *CrysAlis PRO* (Oxford Diffraction, 2009 ▶); cell refinement: *CrysAlis PRO*; data reduction: *CrysAlis PRO*; program(s) used to solve structure: *SHELXS97* (Sheldrick, 2008 ▶); program(s) used to refine structure: *SHELXL97* (Sheldrick, 2008 ▶); molecular graphics: *DIAMOND* (Crystal Impact, 2007 ▶); software used to prepare material for publication: *SHELXL97*.

## Supplementary Material

Crystal structure: contains datablock(s) I, global, New_Global_Publ_Block. DOI: 10.1107/S1600536812021836/rz2749sup1.cif

Structure factors: contains datablock(s) I. DOI: 10.1107/S1600536812021836/rz2749Isup2.hkl

Additional supplementary materials:  crystallographic information; 3D view; checkCIF report

## Figures and Tables

**Table 1:** Hydrogen-bond geometry (Å, °)

*D*—H⋯*A*	*D*—H	H⋯*A*	*D*⋯*A*	*D*—H⋯*A*
O1—H1*O*1⋯F8^i^	0.85	1.92	2.717 (4)	156
O1—H1*O*1⋯F3^i^	0.85	2.23	3.024 (4)	155
O2—H1*O*2⋯F12^ii^	0.87	2.08	2.942 (6)	172
O2—H1*O*2⋯F4^ii^	0.87	2.09	2.949 (6)	172
O2—H1*O*2⋯F6^ii^	0.87	2.31	3.146 (6)	163
O2—H2*O*2⋯F2^iii^	0.85	1.41	2.251 (8)	167
O2—H2*O*2⋯F9^iii^	0.85	1.70	2.491 (8)	153
O2—H2*O*2⋯F7^iii^	0.85	2.00	2.808 (8)	157

Symmetry codes: (i) 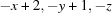; (ii) 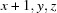; (iii) 
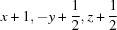
.

**Fig. 1.**
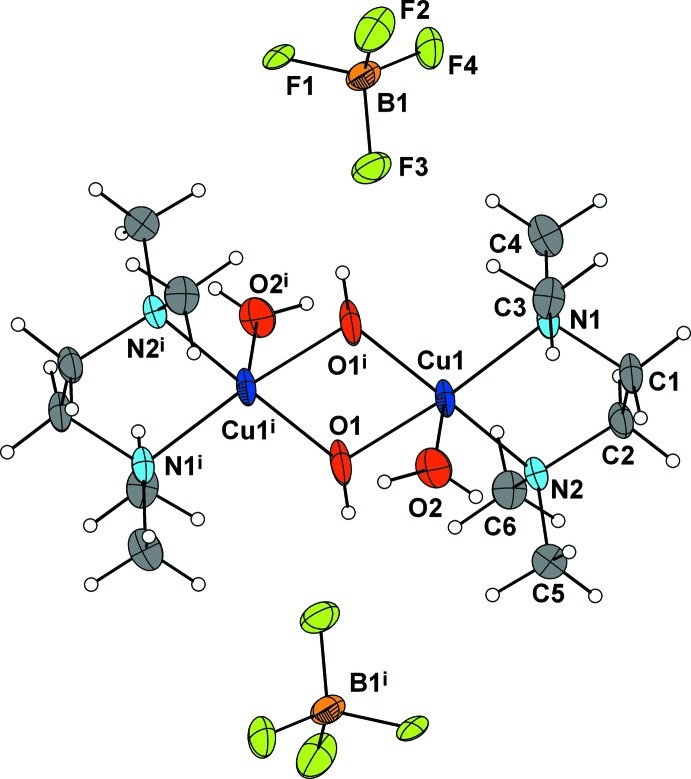
The molecular structure of the title compound. Only the major component of the disordered tetrafluridoborate anion is shown. Displacement ellipsoids are drawn at the 30 % probability level. Symmetry code: (i) 2-x, 1-y, -z.

**Fig. 2.**
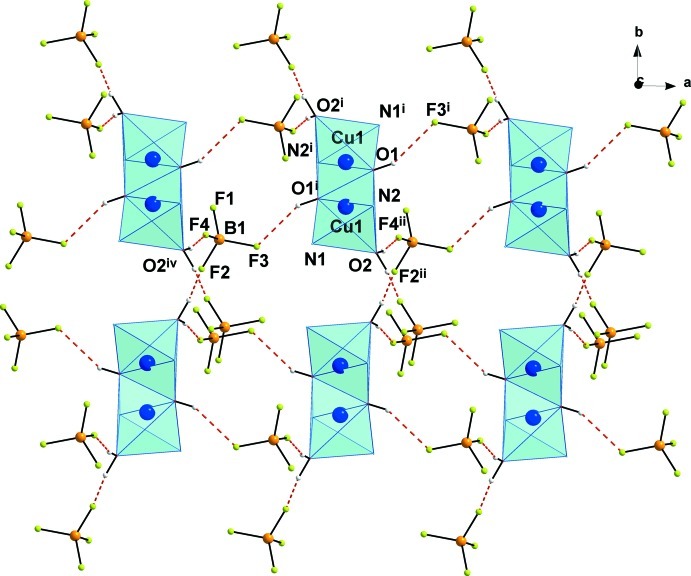
Hydrogen bonding network in the title compound. Only the major components of the tetrafluridoborate anions are shown. Hydrogen bonds are depicted by dashed lines. Symmetry codes: (i) 2-x, 1-y, -z; (ii) 1+x, y, z.

## supplementary crystallographic information

### Comment

As a continuation of our study on crystal structures and magnetic properties of Cu(II) complexes with general formula Cu(*L*–*L*)_2_*X*_2_ (*L*–*L* are *en* or its *N*-methylated derivates, *X* are anions based on fluorine atom; Haníková *et al.*, 2012; Černák *et al.*, 2010) herein we report the crystal structure of the title complex [{Cu(H_2_O)_0.5_(OH)(tmen)}_2_](BF_4_)_2_ (tmen is *N*,*N*,*N*',*N*'-tetramethyl-ethane-1,2-diamine). In contrast to the previously studied complexes Cu(*L*–*L*)_2_*X*_2_ in which two diamine ligands *L*–*L* were coordinated to the Cu(II) atom, in the title complex only one tmen ligand is coordinated, presumably due to steric effects. The crystal structure is essentially ionic and is built up of the centrosymmetric dimeric complex cations and tetrafluoridoborate anions (Fig. 1). In the centrosymmetric complex cation the Cu(II) atom is at 50% pentacoordinated. The calculated value of the τ parameter (τ = 16.4%; Addison *et al.*, 1984) indicates that the coordination polyhedron is close to tetragonal pyramid. On the other hand, in the similar [{Cu(OH)(tmen)}_2_](2BF_4_)_2_ complex the Cu(II) atom exhibits square coordination (Handley *et al.*, 2001). The two µ_2_-hydroxido ligands link the Cu(II) atoms somewhat unsymmetrically, one Cu—O distance being 1.905 (3) Å and the the other 1.916 (3) Å. The coordination plane is completed by two nitrogen atoms from a chelate tmen ligand and the apical position is statistically (50%) occupied by the O atom of a disordered water molecule at a longer Cu—O distance of 2.440 (6) Å. The BF_4_^-^ anion exhibits a high degree of disorder, the fluorine atoms occupying three disordered positions with refined s.o.f.'s of 0.598 (9):0.269 (6):0.138 (8). The crystal structure (Fig. 2) is additionally stabilized by O—H···F hydrogen bonds (Table 1).

### Experimental

The dropwise addition of tmen (0.27 cm^3^, 5 mmol) to a blue solution of Cu(BF_4_)_2_.6H_2_O (1.2 g, 5 mmol) in 20 cm^3^ of a water:ethanol mixture (1:1 *v*/*v*) yielded a violet solution, which was filtered. After seven days blue prisms appeared ion slow evaporation of the solvent which were separated by filtration and dried on air. Yield: 0.55 g (75%).

Anal. [%] (CHNOS Elemental Analyzer vario MICRO, Elementar Analysensysteme GmbH, copper complexometrically) calculated for Cu_2_C_12_N_4_H_36_B_2_F_8_O_3_ (Mr = 585.32): C, 24.63; H, 6.20; N, 9.57; Cu, 21.75. Found: C, 24.70; H, 6.29; N, 9.59; Cu, 22.03. IR (KBr pellets technique, FT–IR Avatar 330 (ThermoNicolet), in cm^-1^): 3633 s; 3547 w; 3408 vs; 3005 w; 2922 *m*; 2902 *m*; 2860 w; 2816 w; 1622 s; 1475 vs; 1084 vs; 951 s; 807 s; 769 *m*; 521 s.

The electronic spectrum (Specord 250 spectrometer, Analytic Jena, in Nujol suspension) of the title compound displays a broad absorption band centred at 567 nm which may represent an envelope of transitions from components of *t*_2g_ to *x*^2^–y^2^ level.

### Refinement

The hydroxy and water H atoms were located in a difference Fourier map and allowed to ride on their parent oxygen atoms with O—H = 0.85 Å and *U*_iso_(H) = 1.5*U*_eq_(O). The positions of all other H atoms were calculated using an appropriate riding model with C—H = 0.98 Å and *U*_iso_(H) = 1.2*U*_eq_(C) (methylene groups) or 1.5*U*_eq_(C) (methyl groups). The fluorine atoms of the anion are disordered over three orientations with refined occupancies of 0.598 (9):0.269 (6):0.138 (8). In order to keep the geometric parameters of the anion chemically reasonable, during the refinement the B–F and F···F distances were constrained to be 1.39 (1) and 2.28 (1) Å, respectively. The fluorine atoms of the lowest contributors were refined isotropically.

### Crystal data

**Table:**

[Cu_2_(OH)_2_(C_6_H_16_N_2_)_2_(H_2_O)](BF_4_)_2_	*F*(000) = 600
*M**_r_* = 585.32	*D*_x_ = 1.615 Mg m^−^^3^
Monoclinic, *P*2_1_/*c*	Mo *K*α radiation, λ = 0.71073 Å
Hall symbol: -P 2ybc	Cell parameters from 3802 reflections
*a* = 7.5878 (3) Å	θ = 2.8–31.9°
*b* = 14.7081 (3) Å	µ = 1.85 mm^−^^1^
*c* = 11.4164 (3) Å	*T* = 103 K
β = 109.174 (4)°	Prism, blue
*V* = 1203.41 (6) Å^3^	0.45 × 0.34 × 0.25 mm
*Z* = 2	

### Data collection

**Table:**

Oxford Diffraction Xcalibur Sapphire2 diffractometer	2118 independent reflections
Radiation source: fine-focus sealed tube	1916 reflections with *I* > 2σ(*I*)
Graphite monochromator	*R*_int_ = 0.017
Detector resolution: 8.3438 pixels mm^-1^	θ_max_ = 25.0°, θ_min_ = 2.8°
ω scans	*h* = −9→6
Absorption correction: numerical [Clark & Reid (1995) in *CrysAlis PRO* (Oxford Diffraction, 2009)]	*k* = −17→17
*T*_min_ = 0.475, *T*_max_ = 0.630	*l* = −12→13
9405 measured reflections	

### Refinement

**Table:**

Refinement on *F*^2^	Primary atom site location: structure-invariant direct methods
Least-squares matrix: full	Secondary atom site location: difference Fourier map
*R*[*F*^2^ > 2σ(*F*^2^)] = 0.046	Hydrogen site location: inferred from neighbouring sites
*wR*(*F*^2^) = 0.143	H-atom parameters constrained
*S* = 1.12	*w* = 1/[σ^2^(*F*_o_^2^) + (0.0802*P*)^2^ + 2.3095*P*] where *P* = (*F*_o_^2^ + 2*F*_c_^2^)/3
2118 reflections	(Δ/σ)_max_ < 0.001
168 parameters	Δρ_max_ = 1.01 e Å^−^^3^
1 restraint	Δρ_min_ = −0.61 e Å^−^^3^

### Special details

**Table:**

Geometry. All e.s.d.'s (except the e.s.d. in the dihedral angle between two l.s. planes) are estimated using the full covariance matrix. The cell e.s.d.'s are taken into account individually in the estimation of e.s.d.'s in distances, angles and torsion angles; correlations between e.s.d.'s in cell parameters are only used when they are defined by crystal symmetry. An approximate (isotropic) treatment of cell e.s.d.'s is used for estimating e.s.d.'s involving l.s. planes.
Refinement. Refinement of *F*^2^ against ALL reflections. The weighted *R*-factor *wR* and goodness of fit *S* are based on *F*^2^, conventional *R*-factors *R* are based on *F*, with *F* set to zero for negative *F*^2^. The threshold expression of *F*^2^ > σ(*F*^2^) is used only for calculating *R*-factors(gt) *etc*. and is not relevant to the choice of reflections for refinement. *R*-factors based on *F*^2^ are statistically about twice as large as those based on *F*, and *R*- factors based on ALL data will be even larger.

### Fractional atomic coordinates and isotropic or equivalent isotropic displacement parameters (Å^2^)

**Table:**

	*x*	*y*	*z*	*U*_iso_*/*U*_eq_	Occ. (<1)
Cu1	0.99395 (7)	0.44147 (3)	0.10450 (4)	0.0366 (2)	
O1	1.1407 (5)	0.5349 (2)	0.0666 (3)	0.0549 (9)	
H1O1	1.2395	0.5509	0.1238	0.066*	
N1	0.8191 (5)	0.3470 (2)	0.1312 (3)	0.0327 (7)	
N2	1.1260 (5)	0.4391 (2)	0.2902 (3)	0.0314 (7)	
C1	0.9128 (7)	0.3110 (3)	0.2589 (4)	0.0415 (10)	
H1A	0.8201	0.2802	0.2892	0.050*	
H1B	1.0090	0.2659	0.2574	0.050*	
C2	1.0012 (6)	0.3876 (3)	0.3435 (4)	0.0409 (10)	
H2A	0.9036	0.4284	0.3536	0.049*	
H2B	1.0744	0.3635	0.4261	0.049*	
C3	0.7852 (7)	0.2687 (3)	0.0449 (4)	0.0439 (10)	
H3A	0.6988	0.2261	0.0637	0.066*	
H3B	0.9035	0.2378	0.0545	0.066*	
H3C	0.7308	0.2904	−0.0406	0.066*	
C4	0.6393 (7)	0.3896 (3)	0.1180 (4)	0.0477 (11)	
H4A	0.5790	0.4079	0.0315	0.072*	
H4B	0.6593	0.4433	0.1716	0.072*	
H4C	0.5594	0.3462	0.1420	0.072*	
C5	1.3102 (6)	0.3950 (3)	0.3208 (4)	0.0418 (10)	
H5A	1.3692	0.3928	0.4110	0.063*	
H5B	1.3892	0.4299	0.2842	0.063*	
H5C	1.2947	0.3331	0.2873	0.063*	
C6	1.1527 (6)	0.5319 (3)	0.3457 (4)	0.0443 (10)	
H6A	1.0323	0.5634	0.3223	0.066*	
H6B	1.2393	0.5662	0.3149	0.066*	
H6C	1.2041	0.5271	0.4362	0.066*	
O2	1.1575 (9)	0.3292 (5)	0.0210 (6)	0.0530 (17)	0.50
H1O2	1.1982	0.3370	−0.0410	0.079*	0.50
H2O2	1.2127	0.2821	0.0596	0.079*	0.50
B1	0.3609 (3)	0.36611 (14)	−0.2852 (2)	0.0427 (11)	
F1	0.3327 (3)	0.44656 (14)	−0.3535 (2)	0.0314 (13)	0.598 (9)
F2	0.2736 (3)	0.29431 (14)	−0.3627 (2)	0.073 (2)	0.598 (9)
F3	0.5517 (3)	0.34845 (14)	−0.2352 (2)	0.0541 (16)	0.598 (9)
F4	0.2853 (3)	0.37442 (14)	−0.1896 (2)	0.063 (2)	0.598 (9)
F5	0.2794 (3)	0.43234 (14)	−0.3741 (2)	0.046 (4)	0.269 (6)
F6	0.2286 (3)	0.33315 (14)	−0.2353 (2)	0.118 (9)	0.269 (6)
F7	0.4266 (3)	0.29504 (14)	−0.3404 (2)	0.114 (7)	0.269 (6)
F8	0.5095 (3)	0.40444 (14)	−0.1910 (2)	0.057 (3)	0.269 (6)
F9	0.2163 (3)	0.32255 (14)	−0.3763 (2)	0.103 (14)*	0.134 (8)
F10	0.5273 (3)	0.31908 (14)	−0.2676 (2)	0.046 (7)*	0.134 (8)
F11	0.3789 (3)	0.45493 (14)	−0.3227 (2)	0.024 (6)*	0.134 (8)
F12	0.3210 (3)	0.36779 (14)	−0.1743 (2)	0.067 (10)*	0.134 (8)

### Atomic displacement parameters (Å^2^)

**Table:**

	*U*^11^	*U*^22^	*U*^33^	*U*^12^	*U*^13^	*U*^23^
Cu1	0.0540 (4)	0.0325 (3)	0.0289 (3)	−0.0106 (2)	0.0212 (2)	0.00740 (18)
O1	0.082 (2)	0.0584 (19)	0.0257 (15)	−0.0393 (18)	0.0190 (15)	0.0040 (14)
N1	0.0516 (19)	0.0269 (16)	0.0236 (15)	−0.0095 (14)	0.0178 (14)	−0.0008 (13)
N2	0.0404 (17)	0.0258 (17)	0.0333 (17)	−0.0060 (13)	0.0193 (14)	0.0025 (12)
C1	0.061 (3)	0.037 (2)	0.030 (2)	−0.0131 (19)	0.0200 (19)	0.0049 (17)
C2	0.049 (2)	0.048 (2)	0.0278 (19)	−0.015 (2)	0.0164 (17)	0.0039 (18)
C3	0.067 (3)	0.028 (2)	0.038 (2)	−0.0014 (19)	0.019 (2)	−0.0051 (17)
C4	0.064 (3)	0.039 (2)	0.051 (3)	−0.007 (2)	0.034 (2)	−0.011 (2)
C5	0.045 (2)	0.038 (2)	0.044 (2)	−0.0001 (18)	0.0163 (19)	−0.0053 (19)
C6	0.056 (3)	0.032 (2)	0.046 (2)	−0.0030 (19)	0.018 (2)	−0.0064 (19)
O2	0.058 (4)	0.077 (5)	0.038 (3)	0.004 (3)	0.034 (3)	−0.006 (3)
B1	0.035 (2)	0.040 (3)	0.044 (3)	0.004 (2)	0.000 (2)	0.006 (2)
F1	0.028 (2)	0.026 (2)	0.024 (2)	−0.007 (2)	−0.013 (2)	−0.0041 (18)
F2	0.088 (4)	0.046 (3)	0.057 (3)	−0.019 (3)	−0.013 (3)	0.005 (2)
F3	0.040 (3)	0.052 (3)	0.060 (3)	0.013 (2)	0.003 (2)	0.011 (3)
F4	0.065 (3)	0.085 (5)	0.051 (3)	0.007 (3)	0.033 (3)	0.029 (3)
F5	0.041 (6)	0.042 (6)	0.037 (6)	−0.005 (5)	−0.010 (5)	−0.003 (4)
F6	0.059 (8)	0.043 (8)	0.26 (3)	0.002 (6)	0.067 (12)	0.022 (11)
F7	0.042 (7)	0.060 (8)	0.24 (2)	−0.005 (6)	0.046 (10)	−0.061 (11)
F8	0.039 (5)	0.084 (9)	0.041 (6)	0.006 (6)	0.001 (4)	0.004 (6)

### Geometric parameters (Å, º)

**Table:**

Cu1—O1	1.905 (3)	C4—H4B	0.9800
Cu1—O1^i^	1.916 (3)	C4—H4C	0.9800
Cu1—N1	2.013 (3)	C5—H5A	0.9800
Cu1—N2	2.026 (3)	C5—H5B	0.9800
Cu1—O2	2.440 (6)	C5—H5C	0.9800
Cu1—Cu1^i^	2.9683 (8)	C6—H6A	0.9800
O1—Cu1^i^	1.916 (3)	C6—H6B	0.9800
O1—H1O1	0.8499	C6—H6C	0.9800
N1—C4	1.463 (6)	O2—H1O2	0.8678
N1—C3	1.482 (5)	O2—H2O2	0.8533
N1—C1	1.493 (5)	B1—F1	1.3940
N2—C5	1.475 (5)	B1—F7	1.3942
N2—C2	1.489 (5)	B1—F6	1.3942
N2—C6	1.491 (5)	B1—F3	1.3950
C1—C2	1.492 (6)	B1—F11	1.3950
C1—H1A	0.9900	B1—F10	1.3950
C1—H1B	0.9900	B1—F4	1.3953
C2—H2A	0.9900	B1—F12	1.3954
C2—H2B	0.9900	B1—F9	1.3956
C3—H3A	0.9800	B1—F5	1.3960
C3—H3B	0.9800	B1—F8	1.3964
C3—H3C	0.9800	B1—F2	1.3969
C4—H4A	0.9800		
			
O1—Cu1—O1^i^	78.06 (15)	H3A—C3—H3B	109.5
O1—Cu1—N1	174.71 (14)	N1—C3—H3C	109.5
O1^i^—Cu1—N1	97.06 (13)	H3A—C3—H3C	109.5
O1—Cu1—N2	97.18 (13)	H3B—C3—H3C	109.5
O1^i^—Cu1—N2	170.09 (14)	N1—C4—H4A	109.5
N1—Cu1—N2	87.33 (12)	N1—C4—H4B	109.5
O1—Cu1—O2	89.4 (2)	H4A—C4—H4B	109.5
O1^i^—Cu1—O2	83.9 (2)	N1—C4—H4C	109.5
N1—Cu1—O2	92.11 (19)	H4A—C4—H4C	109.5
N2—Cu1—O2	104.89 (18)	H4B—C4—H4C	109.5
O1—Cu1—Cu1^i^	39.16 (10)	N2—C5—H5A	109.5
O1^i^—Cu1—Cu1^i^	38.90 (9)	N2—C5—H5B	109.5
N1—Cu1—Cu1^i^	135.92 (9)	H5A—C5—H5B	109.5
N2—Cu1—Cu1^i^	135.73 (9)	N2—C5—H5C	109.5
O2—Cu1—Cu1^i^	85.68 (16)	H5A—C5—H5C	109.5
Cu1—O1—Cu1^i^	101.94 (15)	H5B—C5—H5C	109.5
Cu1—O1—H1O1	117.4	N2—C6—H6A	109.5
Cu1^i^—O1—H1O1	140.6	N2—C6—H6B	109.5
C4—N1—C3	108.2 (3)	H6A—C6—H6B	109.5
C4—N1—C1	112.5 (3)	N2—C6—H6C	109.5
C3—N1—C1	107.2 (3)	H6A—C6—H6C	109.5
C4—N1—Cu1	109.1 (3)	H6B—C6—H6C	109.5
C3—N1—Cu1	114.5 (3)	Cu1—O2—H1O2	126.3
C1—N1—Cu1	105.5 (2)	Cu1—O2—H2O2	125.0
C5—N2—C2	111.1 (3)	H1O2—O2—H2O2	106.4
C5—N2—C6	108.4 (3)	F7—B1—F6	109.6
C2—N2—C6	108.0 (3)	F1—B1—F3	109.6
C5—N2—Cu1	111.1 (3)	F11—B1—F10	109.5
C2—N2—Cu1	105.9 (2)	F1—B1—F4	109.6
C6—N2—Cu1	112.3 (3)	F3—B1—F4	109.5
C2—C1—N1	109.4 (3)	F11—B1—F12	109.5
C2—C1—H1A	109.8	F10—B1—F12	109.5
N1—C1—H1A	109.8	F11—B1—F9	109.5
C2—C1—H1B	109.8	F10—B1—F9	109.5
N1—C1—H1B	109.8	F12—B1—F9	109.4
H1A—C1—H1B	108.2	F7—B1—F5	109.5
N2—C2—C1	109.3 (3)	F6—B1—F5	109.5
N2—C2—H2A	109.8	F7—B1—F8	109.4
C1—C2—H2A	109.8	F6—B1—F8	109.5
N2—C2—H2B	109.8	F5—B1—F8	109.3
C1—C2—H2B	109.8	F1—B1—F2	109.5
H2A—C2—H2B	108.3	F3—B1—F2	109.3
N1—C3—H3A	109.5	F4—B1—F2	109.3
N1—C3—H3B	109.5		
			
O1^i^—Cu1—O1—Cu1^i^	0.0	O2—Cu1—N2—C5	17.2 (3)
N2—Cu1—O1—Cu1^i^	−171.19 (16)	Cu1^i^—Cu1—N2—C5	−82.1 (3)
O2—Cu1—O1—Cu1^i^	83.9 (2)	O1—Cu1—N2—C2	165.2 (3)
O1^i^—Cu1—N1—C4	−65.4 (3)	N1—Cu1—N2—C2	−12.1 (3)
N2—Cu1—N1—C4	105.7 (3)	O2—Cu1—N2—C2	−103.5 (3)
O2—Cu1—N1—C4	−149.5 (3)	Cu1^i^—Cu1—N2—C2	157.2 (2)
Cu1^i^—Cu1—N1—C4	−63.6 (3)	O1—Cu1—N2—C6	47.5 (3)
O1^i^—Cu1—N1—C3	55.9 (3)	N1—Cu1—N2—C6	−129.7 (3)
N2—Cu1—N1—C3	−133.0 (3)	O2—Cu1—N2—C6	138.8 (3)
O2—Cu1—N1—C3	−28.2 (3)	Cu1^i^—Cu1—N2—C6	39.5 (3)
Cu1^i^—Cu1—N1—C3	57.8 (3)	C4—N1—C1—C2	−78.2 (4)
O1^i^—Cu1—N1—C1	173.6 (3)	C3—N1—C1—C2	163.0 (4)
N2—Cu1—N1—C1	−15.3 (3)	Cu1—N1—C1—C2	40.6 (4)
O2—Cu1—N1—C1	89.5 (3)	C5—N2—C2—C1	−83.0 (4)
Cu1^i^—Cu1—N1—C1	175.4 (2)	C6—N2—C2—C1	158.3 (4)
O1—Cu1—N2—C5	−74.1 (3)	Cu1—N2—C2—C1	37.7 (4)
N1—Cu1—N2—C5	108.7 (3)	N1—C1—C2—N2	−53.7 (5)

Symmetry code: (i) −*x*+2, −*y*+1, −*z*.

### Hydrogen-bond geometry (Å, º)

**Table:**

*D*—H···*A*	*D*—H	H···*A*	*D*···*A*	*D*—H···*A*
O1—H1*O*1···F8^i^	0.85	1.92	2.717 (4)	156
O1—H1*O*1···F3^i^	0.85	2.23	3.024 (4)	155
O2—H1*O*2···F12^ii^	0.87	2.08	2.942 (6)	172
O2—H1*O*2···F4^ii^	0.87	2.09	2.949 (6)	172
O2—H1*O*2···F6^ii^	0.87	2.31	3.146 (6)	163
O2—H2*O*2···F2^iii^	0.85	1.41	2.251 (8)	167
O2—H2*O*2···F9^iii^	0.85	1.70	2.491 (8)	153
O2—H2*O*2···F7^iii^	0.85	2.00	2.808 (8)	157

Symmetry codes: (i) −*x*+2, −*y*+1, −*z*; (ii) *x*+1, *y*, *z*; (iii) *x*+1, −*y*+1/2, *z*+1/2.
